# Psychological Distress in Breast Cancer Patients during the Italian COVID-19 Pandemic

**DOI:** 10.3390/ijerph191811433

**Published:** 2022-09-11

**Authors:** Maria Rosa Stanizzo, Lorys Castelli, Cristina Di Nardo, Monica Brunetti, Corrado De Sanctis, Ada Ghiggia

**Affiliations:** 1 Breast Unit, Department of Gynecology and Obstetrics, AOU Città della Salute e della Scienza, 10126 Turin, Italy; 2 Clinical Psychology Unit, AOU Città della Salute e della Scienza, 10126 Turin, Italy; 3 Department of Psychology, University of Turin, 10124 Turin, Italy; 4 Department of Life Sciences, University of Trieste, 34128 Trieste, Italy

**Keywords:** breast cancer, psycho-oncology, COVID-19, post-traumatic stress symptoms, psychological distress

## Abstract

Background. The emergency caused by the SARS-CoV-2 pandemic exacerbated psychological distress. Our aim was to investigate the impact of breast cancer on patients’ lives during the Italian lockdown. Methods. Sixty-five female breast cancer patients were studied, assessing the level of psychological distress with the Hospital Anxiety and Depression Scale (HADS) and the impact of the cancer diagnosis (Impact of Event Scale-Revised—IES—R). In addition, we compared these data with a matched group of breast cancer patients enrolled in 2019. Results. Patients enrolled in 2020 had statistically higher levels of anxious symptomatology and higher levels of traumatic symptomatology due to the cancer diagnosis. A mediation analysis was performed to determine how the experience of distress due to COVID-19 negatively impacted the level of anxiety and amplified the impact of the diagnosis with a significant increase in traumatic symptoms. Conclusions. Considering the vulnerability of these patients and the serious and novel situation that the healthcare system is currently facing, we would like to point out the importance of structured and organised psychological support for these patients.

## 1. Introduction

Italy was the first European country to be affected by the COVID-19 outbreak. The National Health System had to reallocate its resources from elective and semi-elective patients to severe COVID-19 patients. The severity of this pandemic put great pressure on healthcare systems worldwide: to control the enormous spread of SARS-CoV-2 infection, the Italian government imposed strict national measures, such as restricting the free movement of the population throughout the Italian territory, except when necessary for professional or health reasons. Due to the state of emergency, the closure of all non-emergency outpatient activities was announced, as many hospital departments were specifically assigned to treat COVID-19, and medical professionals were assigned to care for COVID-19 patients. In this context, it was necessary to deny nurses access to hospitals and restrict access to patients. In oncology, concerns have been raised about the risk of SARS-CoV-2 infection associated with hospital visits and admissions for treatment, as well as the potential additional risk associated with chemotherapy-induced immunosuppression [1]. Cancer is considered a life-threatening disease that can cause some degree of disability and involves treatments with potentially severe effects on individuals [2]. In addition to the concerns arising from cancer, it is important to consider that for the cancer population, there are additional concerns related to the risks of infection with COVID-19 and the risks arising from postponement and cancelation of already scheduled treatments and screening programs [3]. Therefore, cancer patients have been defined as those who need the most attention in terms of prevention and care during the COVID-19 emergency [4].

In addition, it should be considered that having a person from whom and with whom one receives support to share emotions and thoughts, discuss, decide, and manage visits and care is a resource [2]. Social support is known to be associated with lower levels of depression [5]. The social isolation perceived as a result of access restrictions for family caregivers to the hospital is only one side of the coin. In clinical practice, it has been shown that cancer patients and caregivers limit mutual contact even in daily routines to minimize the risk of possible infection. Our hypothesis is that the emergency situation caused by the pandemic has exacerbated psychological distress, thereby increasing the stressful impact of cancer on patients’ lives. While previous studies have shown that approximately 30–40% of breast cancer patients suffer from depression, anxiety, or adjustment disorders [6], we must consider that the restrictions imposed by the COVID-19 pandemic may have increased the level of perceived anxiety [7].

To test our hypothesis, we compared two groups of cancer patients to examine the impact of the pandemic on individuals who received a breast cancer diagnosis during the COVID-19 emergency. We then compared the 2020 group to a control group of breast cancer patients who had been diagnosed and tested one year earlier (2019) to determine whether the experience of exposure to COVID-19 constraints negatively impacted the level of anxiety symptomatology and led to a significant increase in traumatic symptoms.

## 2. Materials and Methods

### 2.1. Sample and Procedure

Seventy-one female patients were consecutively enrolled in the study from March to May 2020. The final sample consisted of 65 patients with breast cancer (2020 group). Participants were female, had a diagnosis of breast cancer, were at least 18 years of age, could read and understand sufficient Italian, and had no current psychiatric diagnoses or cognitive deficits that would prevent them from providing valid informed consent.

Considering the high number of female participants, also including male participants in the research could have given us unbalanced subgroups and could have created sexual bias in the data.

Nurses and psychologists administered the Hospital Anxiety and Depression Scale (HADS) and the Impact of Event Scale-Revised (IES-R) to patients waiting for medical examination or consultation. A psychological clinical consultation was then recommended for patients with a score equal to or greater than the cut-off on HADS and/or DT. Sociodemographic data were also collected. Data collection was performed by the Department of Clinical Psychology of the “Città della Salute e della Scienza”, Hospital of Turin. Participants were recruited in the breast unit of Sant’Anna Hospital by a psycho-oncologist from the Clinical Psychology Unit.

For the 2019 group, we selected the same number of patients considering the eligibility criteria. The sample was drawn from data collected in the previous year, from March to May 2019. We selected 65 patients consecutively examined during the selected period to investigate the effect of the pandemic on the impact of diagnosis. Only patients who met the same inclusion criteria of the 2020 group and were screened with the same tests were selected. All participants provided written informed consent, and the present study was approved by the Institutional Ethics Committee.

### 2.2. Measures

The Italian version of the HADS was used for the study [8]. This is a validated rating scale used to assess the anxious and depressive symptoms in patients with medical conditions, and it is divided into two subscales, HADS-A for anxious symptomatology and HADS-D for depressive symptomatology. Each scale has 7 items, for a total of 14 items, on a Likert scale ranging from 0 to 3. The total score ranges from 0 to 2; a score of 8 or more suggests a clinically relevant anxious/depressive symptomatology [9].

The Italian version of the IES-R [10,11], a self-administered questionnaire with 22 items on a 5-point Likert scale (0–4, with labels of ‘Not at all’ to ‘Extremely’), was used to measure post-traumatic stress symptoms (PTSS) related to the experience of cancer. The questionnaire includes three subscales measuring avoidance, intrusion, and hyperarousal, and it was keyed to the experience of having cancer. The total score can be considered normal (0–23) or indicative of mild (24–32), moderate (33–36), or severe (≥37) psychological impact.

### 2.3. Statistical Analysis

Values of asymmetry and kurtosis between −1 and +1 were considered acceptable to prove a normal univariate distribution of the data. According to these criteria, the normality assumption was met for all variables. Means (SD) and frequencies were used for the descriptive analyses. If the variable was continuous, comparisons between the two groups (2019 vs. 2020) were performed using the t test; for categorical data, we used the χ2.

First, a hierarchical multiple regression analysis was used to examine whether age, clinical characteristics (treatment and first diagnosis), time of diagnosis (2019 vs. 2020), and psychological distress (anxiety and depression) significantly contributed to the explanation of post-traumatic stress symptomatology, using the IES-R total score as the outcome variable. Second, a mediation analysis was conducted to test the mediating effect of anxiety symptomatology in the relationship between the year (2019 vs. 2020) and the PTSS (IES-R) after testing the hypotheses [12]. Specifically, we aimed to determine whether the experience of distress due to COVID-19 negatively affected the level of anxiety symptomatology and led to a significant increase in traumatic symptoms. All analyses were performed using SPSS Statistics version 26.0.0 software (IBM Corp. Armonk, NY, USA). The PROCESS macro 3.4.1 for SPSS developed by Andrew F. Hayes (2018) was used to test mediation model. All statistical tests were 2-tailed with a value of 0.05.

## 3. Results

The two groups (2019 and 2020) were identical in terms of age [t(127) = −0.755; *p* = 0.452] and education [χ2(4) = 4.846, *p* = 0.0.303]. More than half of the 2020 sample (58.8%) had children, similar to the 2019 group (75%) [χ2(1) = 3.631, *p* = 0.0.057].

Sociodemographic data and comparisons between groups are shown in Table 1.

In almost all patients tested in 2020, breast cancer was a first diagnosis (87.3%), and they were tested about 2 weeks before surgery; otherwise, only seven cases were cancer recurrence. Similarly, in the 2019 sample, 64.5% of patients received an initial diagnosis, one-third of patients (30.6%) were tested in the weeks after surgery, and 25.8% were tested in the later follow-up periods.

Regarding oncological treatment, the majority of the 2020 patients (88.5%) did not receive treatment during the assessment by the psychologist. Moreover, most of them (83.9%) were not taking psychotropic drugs. In contrast, in the 2019 group, only 34.4% were not on pharmacological treatment, and the others were on treatment (adjuvant chemotherapy, postoperative chemotherapy, or hormone therapy).

Regarding psychological distress, more than half of the 2020 patients (52.5%) had a clinically relevant measure of anxiety symptoms (HADS-A), with a significantly higher mean score in the 2020 group than in the 2019 group (see Table 1). No statistical differences were found between levels of depressive symptomatology on the HADS-D score, with 72.1% of 2020 patients reporting a score above the cut-off score, similar to the 2019 group (69.4%).

During 2020, nearly half of participants (41%) had scores indicating severe symptomatology (i.e., IES-R ≥ 37), whereas in the 2019 group, only 21% of patients reported severe PTSS related to the cancer diagnosis, and 64.5% were in the normal range.

**Figure 1:**
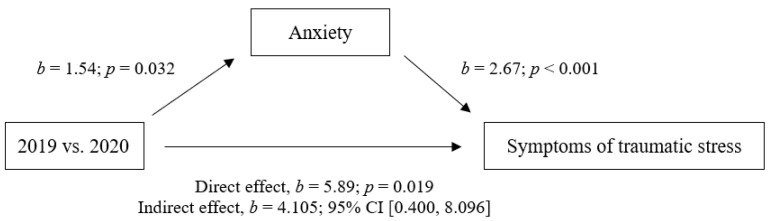
Model of group variable (2019 vs. 2020) as a predictor of traumatic symptoms (IES-R), mediated by anxiety (HADS-Anx). A BCa bootstrapped CI based on 5000 samples was used to measure the confidence interval for the indirect effect.

A preliminary hierarchical multiple regression was performed to examine which of the variables, age, treatment and first diagnosis, the group variable (2019 vs. 2020) and psychological distress (HADS-D and HADS-A), were responsible for significantly higher variance in the IES-R total score. We first introduced age in Model 1 [ΔR^2^= 0.001, F(1,114) = 0.07, *p* = 0.792] and treatment and first diagnosis variables in Model 2 [ΔR^2^= 0.026, F(2,112) = 1.01, *p* = 0.233]. The addition of the group variables (2019 and 2020) in Model 3 [F(1, 111) = 2.57, *p* = 0.042] showed a significant contribution to PTSS (IES-R), with an R^2^ of 0.085 (adjusted R^2^ = 0.052). In the final step, Model 4 [F(2, 109) = 14.81, *p* < 0.001], this effect was not maintained when psychological distress (HADS-D and HADS-A) was also included, with a statistically significant increase in R^2^ of 0.364 (adjusted R^2^ = 0.364). Specifically, the HADS anxiety resulted as the strongest contributing factor (β = −0.522, *p* < 0.001). Based on these results, we hypothesized that anxiety (HADS-A) played a mediating role in the relationship between the 2019 and 2020 groups and the PTSS (IES-R total score). The mediation model confirmed our hypothesis, as a significant indirect effect of 2020 group classification on IES-R was found via HADS-A, b = 4.105, 95% CI [0.400, 8.096] (Figure 1).

## 4. Discussion

Even in the absence of a pandemic, a cancer diagnosis has a strong impact on the individual. In our study, the psychological distress of cancer patients examined in 2020 group was significantly higher than that of a comparison group examined in 2019. 

The most important finding is that almost twice as many patients reported significant PTSS during the pandemic COVID-19 than the comparable patients studied during the same period one year earlier. However, it should also be considered that changes in the degree of psychological distress were described depending on the time of assessment [13], which is due to the different situations usually faced during the different phases of the disease [2,14]. 

Cancer patients live with an already very traumatic disease during a time of stress and anxiety, leading to an exacerbation of post-traumatic stress symptoms. In addition, fear of infection and increased loneliness and demoralization inevitably led patients to become more concerned with pathology and the associated risk of contracting SARS-CoV-2 with possible progression to COVID-19 due to secondary systemic immunosuppression and cancer treatment. Added to the fear of oncologic disease was the fear of infection and the serious consequences, even death. Therefore, limited recommendations were made for patients currently receiving active treatment and for patients in follow-up [15]. In addition, patients had to manage the situation alone. Because of the restrictions, caregivers were not allowed to participate in hospital visits; consequently, they were highly focused on the disease and medical procedures.

While waiting for treatment and visits, patients could not distract themselves from the situation because it was not possible for them to interact with caregivers and volunteers; therefore, it was natural for them to focus more on the experience of the disease and to share their experiences only with patients in the same situation. Patients also had to pay more attention during visits to physicians and medical professionals than before the restrictions imposed by COVID-19. The absence of caregivers with whom they could share the moments of the oncology journey highlighted the need for patients to understand and retain all the information given during visits and examinations, with the consequence that they had to devote all their attention to what was happening.

Recent studies have examined the psychological reactions to the onset of COVID-19 in the general population [16,17]. In particular, the lockdown condition has been associated with significant negative psychological effects [18]. Similarly, oncology patients have experienced the same isolation [19]. People have restructured their priorities and put health first, giving greater importance to aspects of protection from COVID-19 disease than to those related to cancer for fear of infection and its consequences.

It is well known that stress impairs the proper functioning of the immune system and increases the risk of disease [20,21]. Moreover, stress-related psychosocial factors decrease survival rates and thus increase mortality in cancer patients [22,23,24]. Finally, the pandemic emergency required the redistribution of most available healthcare resources for the treatment of COVID-19-positive patients, which in several cases has led to a reduction in direct contact with healthcare workers, exacerbating the lack of social support among patients [25] and increasing stress among healthcare workers themselves [26].

Several limitations should be noted when considering the present study. First, only female patients were selected. Second, although this study provides important information about the psychological consequences of COVID-19 restriction, the cross-sectional design of the study does not allow for causal relationships. Third, some clinical characteristics of the sample may limit the generalizability of the results.

## 5. Conclusions

In summary, despite the cross-sectional nature of the study, this survey highlights the high stress caused by the COVID-19 pandemic in a breast cancer population. Therefore, in an extraordinary emergency, it seems more necessary to provide timely psychological support in lockdown situations (possibly including the use of telemedicine) that can be repeated to reduce the psychological impact on patients already suffering from organic disease and to be aware of the possible development of post-traumatic stress disorder in cancer survivors.

## Figures and Tables

**Table 1:** Socio-demographic and psychological variables in 2020 patients’ group, compared to the 2019 group.

Variables	2020 (n = 65)	2019 (n = 65)	*p*-Value
**Age** (mean ± SD)	59.2 ± 11.1	58.0 ± 11.0	0.452
**Marital status** (%)			0.539
Cohabitant	68.5%	72.9%	
Single	14.8%	8.5%	
Divorced	3.7%	8.5%	
Widowed	13%	10.2%	
**Education** (%)			0.303
Primary education	8.3%	8.1%	
Lower secondary education	26.7%	40.3%	
Upper secondary education	50%	32.3%	
Bachelor or master degree	15%	19.4%	
**Employment** (%)			0.489
Housewife	13.3%	16.7%	
Worker	50.0%	53.3%	
Unemployed	8.3%	3.3%	
Retired	28.3%	25%	
**HADS—total score**, (mean ± SD)	13.48 ± 7.90	11.92 ± 6.67	0.231
HADS—depression	5.45 ± 4.18	5.45 ± 3.84	0.912
HADS—anxiety	8.02 ± 4.36	6.47 ± 3.65	0.032 *
**IES-R—total score**, (mean ± SD)	32.92 ± 18.20	22.70 ± 16.05	0.001 *

* *p* value < 0.05.

## Data Availability

Data are available from the corresponding author upon reasonable request.
